# Studies on the Bioactive Flavonoids Isolated from *Pithecellobium clypearia* Benth

**DOI:** 10.3390/molecules19044479

**Published:** 2014-04-10

**Authors:** Jie Kang, Chao Liu, Hongqing Wang, Baoming Li, Chao Li, Ruoyun Chen, Ailin Liu

**Affiliations:** State Key Laboratory of Bioactive Substance and Function of Natural Medicines, Institute of Materia Medica, Chinese Academy of Medical Sciences & Peking Union Medical College, Beijing 100050, China; E-Mails: jiekang@imm.ac.cn (J.K.); liuchao@imm.ac.cn (C.L.); wanghongqing@imm.ac.cn (H.W.); libaoming@imm.ac.cn (B.L.); lichao880919@imm.ac.cn (C.L.)

**Keywords:** (2*R*,3*R*)-7-*O*-galloylplumbocatechin A, *Pithecellobium clypearia* Benth, neuraminidase (NA) of influenza virus, anti-inflammatory activity

## Abstract

One new flavonoid named (2*R*,3*R*)-7-*O*-galloylplumbocatechin A (**1**) and three known flavonoids, (−)-5,3',4',5'-tetrahydroxyflavan-7-gallate (**2**), (+)-3,5,3',4',5'-penta-hydroxyflavan-7-gallate (**3**), and (−)-7,4'-di-*O*-galloyltricetiflavan (**4**), were isolated from *Pithecellobium clypearia* Benth. Their structures were elucidated based on spectroscopic analysis, including homonuclear and heteronuclear correlation NMR (HSQC and HMBC) experiments. *In vitro* assays, compounds **1** and **2** showed moderate inhibitory effects against influenza H1N1 virus neuraminidase (NA). Compounds **1**–**4** were all found to inhibit the expression of proinflammatory cytokines IL-6 or MCP-1 induced by influenza H1N1 virus in human A549 lung carcinoma cells.

## 1. Introduction

The influenza virus is a highly infective agent that causes acute pulmonary diseases [1,2,3]. The influenza virus contains eight pieces of segmented RNA, with neuraminidases (NAs) as surface antigens. NAs are involved in the release of progeny virus from infected cells, by cleaving sugars that bind the mature viral particles. Specifically, NAs cleave the α-ketosidic bond that links a terminal neuraminic-acid residue to the adjacent oligosaccharide moiety, therefore NAs are essential for the movement of the virus to and from sites of infection in the respiratory tract [4,5].

Recent studies have shown that certain strains of influenza virus, such as H5N1, are more likely to induce excessive cytokine release and immune cell exudation [6,7]. This so-called “cytokine storm” scenario, features elevated levels of cytokines and chemokines such as tumor necrosis factor (TNF)-α, interleukin (IL)-6, and monocyte chemotactic protein (MCP)-1, which causes tissue damage, impairs normal mucosal membrane and may induce airway blockage, making it a risk factor for the higher mortality associated with these virulent strains. Therefore, alleviating inflammation during influenza virus infection could potentially be beneficial [8].

*Pithecellobium clypearia* Benth, a member of Mimosaceae family, is widely distributed in the South of China, such as Sichuan, Yunnan, and Guangdong provinces. *P. clypearia* has been found to contain various flavonoids. It has been used as a herbal medicine in the treatment of respiratory tract diseases in China for many years [9,10,11,12]. One Chinese patent medicine manufactured from the aqueous extract of the leaves and twigs of *P. clypearia* has been recorded in the Pharmacopeia of China, and used for the treatment of upper respiratory tract infections, pharyngitis, laryngitis, and acute tonsillitis, [9,10,11,12]. Though some of flavonoids from *P. clypearia* were reported to have anti-viral activity against H1N1 and herpes simplex [9], the mechanisms underlying the effects of *P. clypearia* have not been identified clearly.

Our previous screening showed that the EtOAc extract of *P. clypearia* exhibited *in vitro* influenza virus neuraminidase (NA)-inhibitory activity, with IC_50_ values of 26.9 ± 1.05 μg/mL. In the present study, the chemical constituents of the EtOAc extract were separated, and their NA inhibitory and anti-inflammatory activities were both evaluated to partially reveal the responsible compounds and the mechanism of action against influenza virus.

## 2. Results and Discussion

### 2.1. Characterisation of Compound **1**

**Figure 1:**
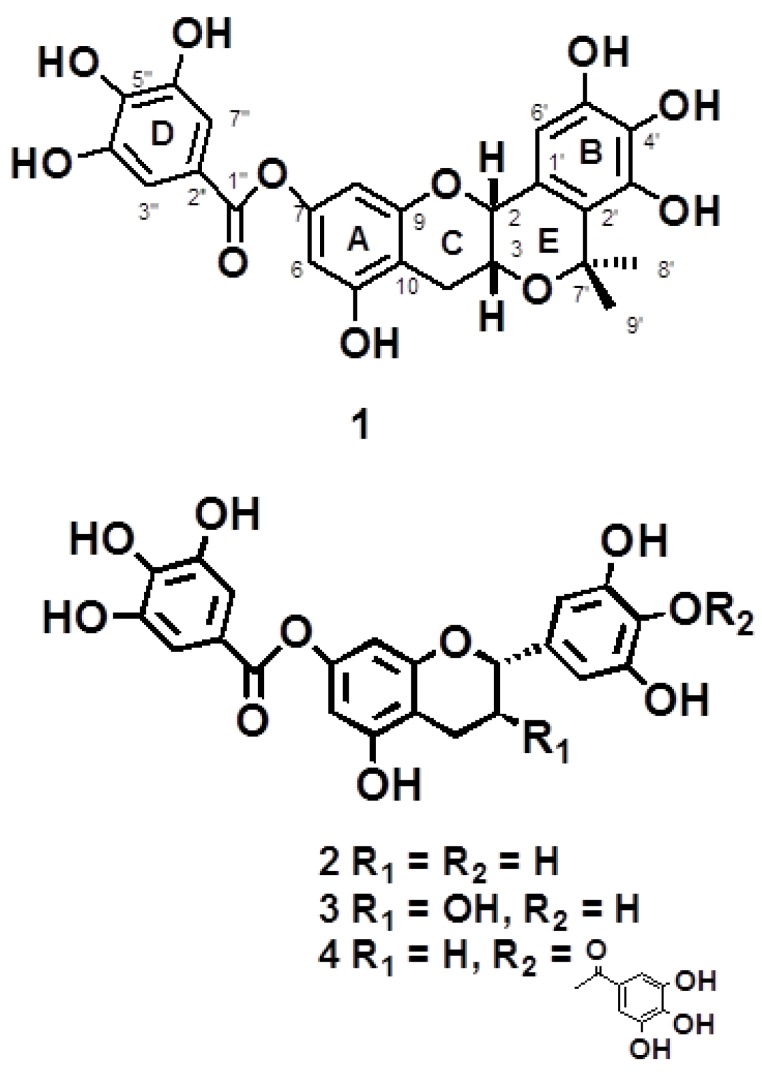
The structures of compounds **1**–**4** isolated from *P. clypearia*.

Four flavonoids (Figure 1) were obtained from *P. clypearia*. Among them, compound **1** is new and its structure was elucidated by HR-ESI-MS, UV, IR, ^1^H- and ^13^C-NMR, HMBC, HMQC, NOE difference and CD spectroscopy.

Compound **1** was obtained as a white amorphous powder, and its molecular formula C_25_H_22_O_11_ was determined from the molecular ion peak at *m*/*z* 499.1224 [M + H]^+^ deduced by HR-ESI-MS. The IR spectrum showed hydroxyl and carbonyl groups at 3189 and 1721 cm^−1^, respectively. IR absorption bands at 1605 and 1515 cm^−1^ for aromatic rings suggested that **1** is likely a phenolic compound.

**Figure 2:**
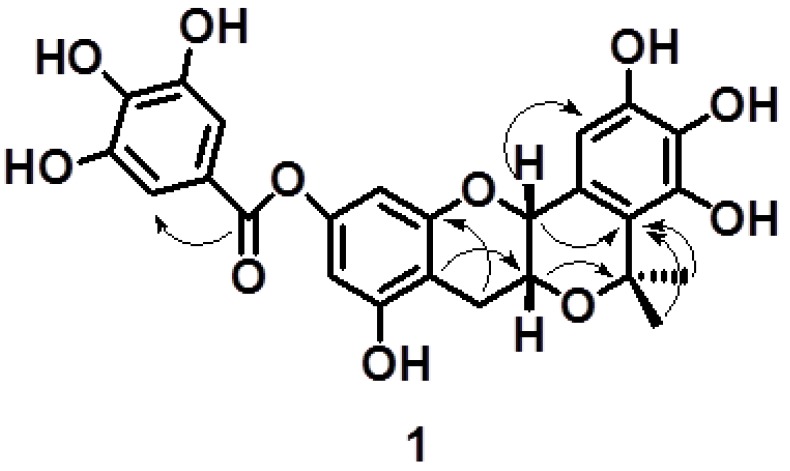
The key HMBC correlations of compound **1**.

The ^1^H-NMR spectrum of **1** exhibited two methyl proton signals at *δ*_H_ 1.51 (3H, s) and 1.45 (3H, s). The aromatic region of the ^1^H-NMR spectrum showed two classic 1,3,4,5-tetrasubstitued aromatic ring patterns [6.00 (1H, s), 6.14 (1H, s); 7.02 (2H, s)], and a pentasubstituted aromatic ring [6.33 (1H, s)]. The other ^1^H-NMR signals at 4.49 (1H, s), 4.17 (1H, d, *J* = 4.4 Hz), 2.83 (1H, dd, *J* = 17.2, 4.4 Hz) and 2.64 (1H, d, *J* = 17.2 Hz) were attributed to H-2, H-3 and H-4 of a flavan nucleus, respectively. The ^13^C-NMR spectrum of compound **1** showed the presence of 25 carbons, including two methyl carbon signals at *δ*_C_ 28.9 and 24.6; one methylene carbon signal at *δ*_C_ 25.8; one signal of carbonyl carbon at *δ*_C_ 164.9; seven methine carbon and fourteen quaternary carbon signals. Compared to that of compound **3** [13], the ^1^H spectrum showed that **1** had two more methyl groups and lacked the signal of H-2' in the B ring. In addition, a relative upfield shift of H-2 (*δ*_H_ 4.49) and downfield shift of H-3 (*δ*_H_ 4.17) of **1** were also observed. The HMBC correlations (^3^*J* = 4.0 Hz) of H-3/C-7' and the HMBC correlations (^3^*J* = 8.0 Hz) of H-2/C-2' and H-8',9'/C-2' (Figure 2) suggested that the hydroxyl group at C-3 was cyclized with the substituent group at C-2'. In comparison with the ^13^C-NMR data of the known compound, (−)-epigallocatechin [14], the C-7 signal was observed to have an upfield shift of about 7.6 ppm, and downfield shifts from 4.8 to 5.4 ppm at C-6, -8 and -10, respectively, which indicated the galloyl ester group of compound **1** was at C-7.

**Figure 3:**
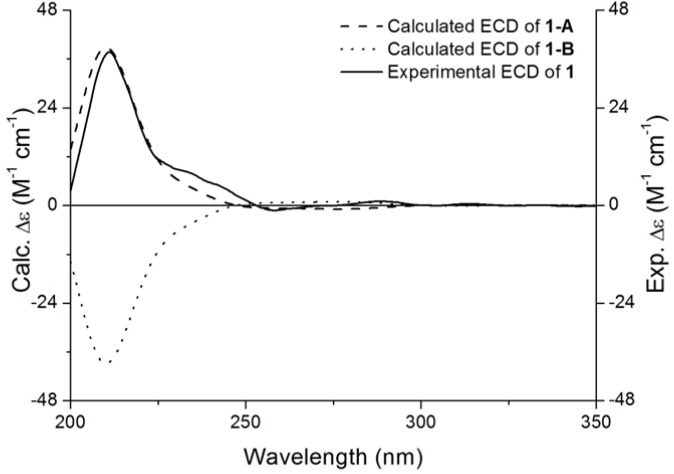
Assignment of the absolute configuration of **1** by comparison between its experimental and calculated CD spectra.

The enhancement of H-2 observed after H-3 was pre-irradiated in a NOE difference experiment, as well as no couplings observed between H-2 and H-3, and H-2 appearing as a singlet, disclosed that the relative configuration of H-2 and H-3 was *cis*. To assign the absolute configuration of **1**, the electronic circular dichroism (ECD) spectrum obtainable through quantum-chemical calculations were used. Along with the result of the NOE difference experiment, there were two possible candidate stereostructures 1-A (2*R*,3*R*) and 1-B (2*S*,3*S*). The ECD spectrum generated for 1-A showing the positive (211 nm) Cotton effect was in good agreement with the experimental data of **1**, whereas the calculated ECD spectrum for 1-B was almost opposite to the experimental curve (Figure 3). The ECD data of 1-A was associated with the experimental Cotton effect at 211 nm. Hence, the structure of new compound **1** was elucidated unambiguously, and it was named (2*R*,3*R*)-7-*O*-galloylplumbocatechin A [15].

### 2.2. NA Inhibitory Effects

Three different subtype NAs were prepared from A/PR/8/34 (H1N1), A/Sydney/5/97 (H3N2) and B/Jiangsu/10/2003. The EtOAc extract of *P. clypearia* exhibited the inhibitory activity on NA from influenza H1N1 virus, with an *IC*_50_ value of 26.9 ± 1.05 μg/mL. Compounds **1** and **2** possessed higher inhibitory activities on NA from influenza H1N1 virus than on those from influenza H3N2 and B viruses (Table 1).

**Table 1:** The flavonoids from *P. clypearia* in this study and their inhibitions on NAs of influenza A and B viruses.

No.	Names		IC_50_ (µg/mL)	
		A/PR/8/34(H1N1)	A/Sydney/5/97(H3N2)	B/Jiangsu/10/2003
1	7-*O*-galloylplumbocatechin A	29.77 ± 6.12	32.23 ± 1.65	39.15 ± 4.67
2	(−)-5,3′,4′,5′-tetrahydroxyflavan-7-gallate	36.91 ± 3.80	>40	>40
3	(+)-3,5,3′,4′,5′-pentahydroxyflavan-7-gallate	>40	>40	>40
4	(−)-7,4′-di-*O*-galloyltricetiflavan	>40	>40	>40
positive control	zanamivir	3.00 × 10^−5^ ± 2.03 × 10^−6^	2.10 × 10^−4^ ± 1.84 × 10^−5^	3.00 × 10^−4^ ± 2.09 × 10^−5^

### 2.3. Inhibition of Cytokine Production in H1N1-Infected Cells

**Figure 4:**
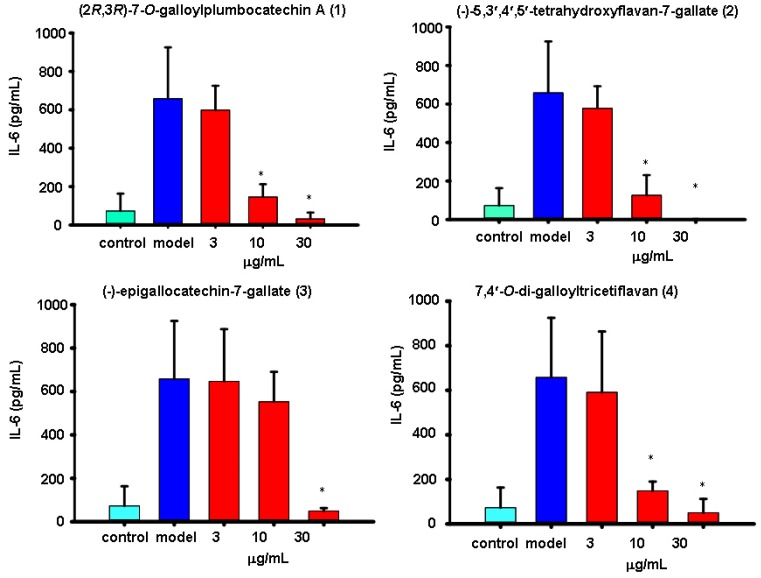
The results of treatment with the four flavonoids **1**–**4** at 3, 10 or 30 μg/mL on the production of IL-6 in H1N1-infected A549 cells. At 24 h post infection, the level of IL-6 in the supernatants was measured by ELISA. Data are presented as the mean ± SD of three separate experiments. * *p* < 0.05compared with model.

**Figure 5:**
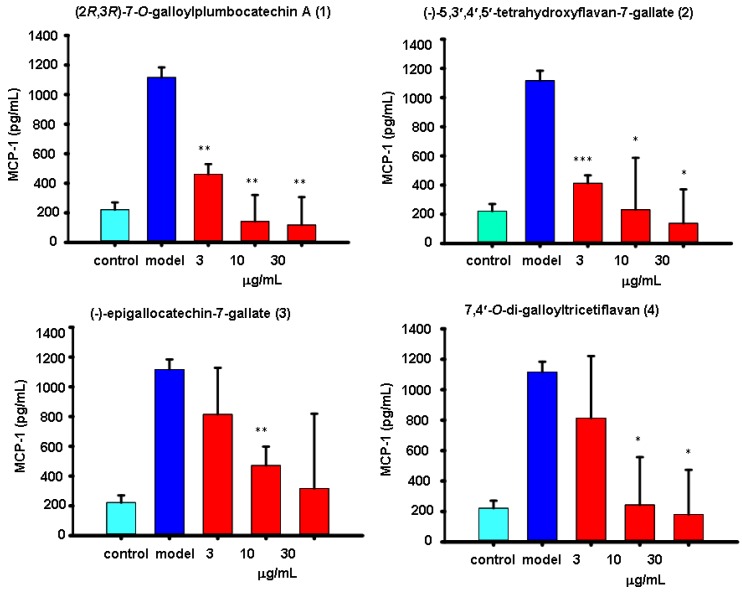
The results of treatment with the four flavonoids **1**–**4** at 3, 10 or 30 μg/mL on the production of MCP-1 in H1N1-infected A549 cells. At 24 h post infection, the level of MCP-1 in the supernatants was measured by ELISA. Data are presented as the mean ± SD of three separate experiments. * *p* < 0.05, ** *p* < 0.01, *** *p* < 0.001 compared with model.

The virus-induced “cytokine storm” appears to contribute to the severe pathogenesis of the reconstructed 1918 H1N1 and H5N1influenza viruses pandemics [16,17]. Therefore, the inhibition of virus-induced cytokine release is also important for the treatment of influenza. Supernatants of human A549 lung carcinoma cells treated or untreated with flavonoids were compared by ELISA for the respective expression levels of IL-6 and MCP-1. Mock-infected cells maintained a level of IL-6 or MCP-1 as low as 73.01 ± 90.31 (Figure 4) or 221.36 pg/mL (Figure 5), while H1N1 infection dramatically increased the level of IL-6 or MCP-1 by 9-fold (657.73 ± 267.43 pg/mL) (Figure 4) or 5-fold (1116.06 ± 67.89 pg/mL) (Figure 5). All four flavonoids screened were found to significantly inhibit the expressions of IL-6 or MCP-1 induced by influenza H1N1 virus at various doses. However, their behaviors were very different. Flavonoids **1**–**4** all showed dose responses in which only higher doses displayed any inhibitory effects against IL-6. Among them, flavonoid **2** showed the best inhibitory effect against IL-6 and flavonoid **3** showed the weakest inhibition (Figure 4). Flavonoids **1**–**2** were both shown to inhibit MCP-1 expression best at the lowest concentrated dilution of 3 μg/mL (Figure 5). One apparent reason for these different results is the difference in chemical structure. Among these four compounds compound **3**, for example, is the only one that bears a hydroxyl group at C-3, and it displayed the worst inhibition of both IL-6 and MCP-1. However, exactly how the chemical structure affects IL-6 or MCP-1 expression and the potent anti-inflammatory effects seen at the lowest dose tested for these compounds still need further investigation.

## 3. Experimental

### 3.1. Plant Material

The leaves and twigs of *P. clypearia* were collected from Shankou, Beihai City, Guangxi Province in May 2012 and were identified by Professor Lin Ma, Department of Natural Products Chemistry, Institute of Materia Medica (IMM), Chinese Academy of Medical Sciences and Peking Union Medical College (CAMS & PUMC), Beijing, China. A voucher specimen (2012032) was deposited in IMM, CAMS & PUMC.

### 3.2. Chemicals and Instruments

Optical rotations were measured with a JASCO P-2000 polarimeter (Tokyo, Japan). UV spectra were collected in MeOH on a JASCO V-650 spectrophotometer (Tokyo, Japan). IR spectra were recorded on a Nicolet 5700 spectrometer (Madison, WI, USA) by the FT-IR transmission electron microscopy method. ^1^H- and ^13^C-NMR spectra were recorded on a MP-400 spectrometer (Palo Alto, CA, USA). HRESIMS were recorded on an Agilent 1200 SL series LC / 6520 QTOF spectrometer (Boleblingen, Germany). Purification was performed using silica gel (160–200 mesh, Qingdao Marine Chemical Factory, Qingdao, China), Sephadex LH-20 (GE Healthcare Bio-Sciences AB, Uppsala, Sweden), and ODS (50 μm, YMC, Kyoto, Japan). Fractions obtained from column chromatography were monitored by TLC (silica gel GF254, Qingdao Marine Chemical Factory, Qingdao, China). 

### 3.3. Cells and Virus Strains

A549 cells (an epithelial cell line through explant culture of lung carcinomatous tissue) were maintained in RPMI1640 medium containing 10% FBS. Before infection, cells were washed with PBS (pH 7.2–7.4) and cultured in media supplemented with 2 μg/mL trypsin TPCK treated from bovine pancreas (Sigma–Aldrich, Saint Lousis, MO, USA, Lot# 031M7358V).

The human influenza virus strains A/PR/8/34 (A/H1N1), A/Sydney/5/97 (H3N2) and B/Jiangsu/10/2003 were kindly donated by the Institute for Viral Disease Control and Prevention, China Centers for Disease Control and Prevention (Beijing, China). Viral stocks of these laboratory-adapted strains were prepared by passaging them in 9-day-old embryonated chicken eggs for 48 or 72 h.

### 3.4. Extraction and Isolation

The leaves and twigs of *P. clypearia* (15 kg) were extracted three times with 95% EtOH (4 L) under reflux. The 95% EtOH extract (2 kg) was subjected to column chromatography over silica gel, eluted with petroleum ether, CHCl_3_, EtOAc, acetone and CH_3_OH. The EtOAc fraction (100 g) was then chromatographed on a silica gel column, with gradient elution (CHCl_3_–MeOH = 9:1 to 1:1). The fraction eluted with CHCl_3_–MeOH = 9:1 was repeatedly chromatographed over a Sephadex LH-20 column eluted with MeOH, then over a C18 column eluted with a linear gradient elution system (30% to 90% MeOH in H_2_O) to yield ten fractions. Repeated chromatography of fraction 2 over Sephadex LH-20 (MeOH) yielded compound **4** (10 mg). Fraction 4 was rechromatographed over Sephadex LH-20, eluted with MeOH to furnish **1** (33 mg) and **2** (28 mg). Rechromatography of fraction 3 on C_18_ using MeOH–H_2_O (from 30% to 90% MeOH) as eluent furnished **3** (20 mg).

### 3.5. Characterization of Compounds **1**–**4**

Compound **1**: white amorphous solid; HR-ESI-MS: *m*/*z* 499.1224 [M + H]^+^, (calcd. for C_25_H_23_O_11_, 499.1235), [*α*]^20^_D_ +90.9 (*c* 0.11, MeOH), UV (MeOH) *λ*_max_: 211, 289 nm, CD (MeOH): 211.0 (∆*ε* +37.74) nm; IR (KBr) *ν*_max_: 3189, 1721, 1605, 1515 cm^−1^. ^1^H- and ^13^C-NMR data, see Table 2. According to the data and reported literature values [13,14,15], compound **1** was identified as (2*R*,3*R*)-7-*O*-galloylplumbocatechin A.

Compound **2**: white amorphous solid; ESI-MS: *m*/*z* 465 [M + Na]^+^, [*α*]^20^_D_ −5.2 (*c* 0.18, MeOH), ^1^H- and ^13^C-NMR data, see Table 2. According to the data and reported literature values [18], compound **2** was determined to be (−)-5,3',4',5'-tetrahydroxyflavan-7-gallate.

Compound **3**: brown amorphous solid; ESI-MS: *m*/*z* 457 [M − H]^−^, [*α*]^20^_D_ +38.4 (*c* 0.17, MeOH), ^1^H- and ^13^C-NMR data, see Table 2. According to the data and reported literature values [13], compound **3** was determined to be (+)-3,5,3',4',5'-pentahydroxyflavan-7-gallate.

Compound **4**: brown amorphous solid; ESI-MS: *m*/*z* 593 [M − H]^−^, [*α*]^20^_D_ −17.9 (*c* 0.12, MeOH), ^1^H- and ^13^C-NMR data, see Table 2. According to the data and reported literature values [19], compound **4** was determined to be (−)-7,4'-di-*O*-galloyltricetiflavan.

Because there had only one chiral center (C-2) in the structures of **2** and **4**, and the optical rotation values of them were both negative, their absolute configuration at C-2 should both be *S* [9,18].

For compound **3**, due to the value of coupling constant (*J*_2,3_ = 6.4 Hz), the relative configuration of H-2 and H-3 should be *trans* [13]. In addition, the optical rotation values of **3** was positive, therefore, the absolute configurations of C-2 and C-3 should be *R* and *S*, respectively [13].

**Table 2:** ^1^H- (400 MHz) and ^13^C-NMR (100 MHz) data of compounds **1**–**4** in DMSO-*d*_6_.

No. position	1	^13^C	2	^13^C	3	^13^C	4	^13^C
	*δ*_H_ (*J* in Hz)		*δ*_H_ (*J* in Hz)		*δ*_H_ (*J* in Hz)		*δ*_H_ (*J* in Hz)	
2	4.49 (s)	71.1	4.78 (d, 9.9)	77.5	4.57 (d, 6.4)	56.8	4.89 (d, 10.4)	76.7
3	4.17 (d, 4.4)	62.4	1.85 (m) 2.02 (m)	29.1	3.89 (m)	81.2	1.86 (m) 2.11 (m)	28.7
4	2.83 (dd17.2, 4.4) 2.64 (d, 17.2)	25.8	2.60 (br s)	19.6	2.66 (dd, 16.4, 4.8) 2.44 (overlapped)	27.1	2.62 (2H, br s)	19.0
5		155.5		156.4		155.1		155.8
6	6.14 (s)	100.9	6.16 (s)	100.9	6.17 (s)	100.4	6.18 (s)	100.6
7		149.9		150.1		149.8		149.7
8	6.00 (s)	101.0	6.09 (s)	101.3	6.11 (s)	100.6	6.13 (s)	100.9
9		156.7		156.5		156.1		156.0
10		104.9		107.4		105.6		107.0
1'		123.2		133.0		132.6		138.7
2'		121.1	6.31 (s)	105.5	6.25 (s)	105.7	6.43 (s)	104.9
3'		144.5		146.4		145.7		150.2
4'		134.3		132.0		129.4		126.4
5'		142.8		146.4		145.7		150.2
6'	6.33 (s)	108.8	6.31 (s)	105.5	6.25 (s)	105.7	6.43 (s)	104.9
7'		75.3						
8'	1.45 (3H, s)	28.9						
9'	1.51 (3H, s)	24.6						
1"		164.9		164.9		164.9		164.5
2"		118.9		118.9		118.4		118.9
3"	7.02 (s)	109.5	7.04 (s)	109.5	7.04 (s)	109.0	7.04 (s)	109.2
4"		146.2		146.2		145.7		145.6
5"		139.6		139.6		139.2		139.2
6"		146.2		146.2		145.7		145.6
7"	7.02 (s)	109.5	7.04 (s)	109.5	7.04 (s)	109.0	7.04 (s)	109.2
1"'								163.9
2"'								118.4
3"'							7.06 (s)	109.0
4"'								145.8
5"'								138.9
6"'								145.8
7"'							7.06 (s)	109.0

### 3.6. Neuraminidase Inhibition Assay

A standard fluorimetric assay was used to measure influenza virus NA activity [19]. The substrate MUNANA is cleaved by NA to yield a fluorescent product which can be quantified. The reaction mixture containing tested extract of *P. clypearia* or compounds **1**–**4**, and NA enzyme or virus suspension in 32.5 mM MES buffer and 4 mM calcium chloride (pH 6.5) was incubated at 37 °C for 40 min. After incubation, the reaction was terminated by adding 34 mM NaOH. Fluorescence was quantified with excitation wavelength at 360 nm and emission wavelength at 450 nm. The 50% inhibitory concentration (IC_50_) was defined as the concentration of NA inhibitor necessary to reduce NA activity by 50% relative to a reaction mixture containing virus but no inhibitor.

### 3.7. Cytokine Secretion Assay

Confluent monolayers of A549 cells in 96-well plates at 37 °C in 5% CO_2_ infected with influenza virus (H1N1) at MOI 0.1 were treated with flavonoids at the serially diluted, non-cytotoxic concentrations of 3, 10 and 30 μg/mL. Supernatants from mock or H1N1-infected cells at 24 h post-infection were compared for the expression of IL-6 and MCP-1 by ELISA (4A Biotech Co. Ltd., Beijing, China) respectively according to the manufacturer’s protocol [20]. The experiments were repeated three times with similar results in three parallel measurements.

### 3.8. Statistical Analysis

All data are given as the mean ± SD. Statistical analysis of the results was performed with Students’ *t*-test. *p* values of <0.05 were considered to be statistically significant.

## 4. Conclusions

The flavonoids are a diverse group of polyphenolic compounds widely distributed in the plant kingdom [21]. In recent years, the scientific community has focused its attention on a class of secondary metabolites—flavonoids—extensively present in a wide range of plant foods, and suggested as having different biological roles [22]. Some flavonoids were reported to display inhibitory activities against NA [23,24,25,26]. Our data confirmed that flavonoids are the major polyphenols in the leaves and twigs of *P. clypearia*. One new flavonoid, (2*R*,3*R*)-7-*O*-galloylplumbocatechin A (**1**) and three known flavonoids, (−)-5,3',4',5'-tetrahydroxyflavan-7-gallate (**2**), (+)-3,5,3',4',5'-pentahydroxyflavan-7-gallate (**3**), (−)-7,4'-di-*O*-galloyltricetiflavan (**4**), were identified from *P. clypearia*.

The virus-induced cytokine response contributes to the activation of the immune system and the damage to the host [27,28]. Suppression of these cytokines can potentially control the severity of the virus-induced inflammatory complications and ultimately lower the mortality [27,28]. These findings provide a possibility that an agent with antiviral and anti-inflammatory activities can be a drug of choice for the treatment of patients with severe influenza-associated complications. A certain types of flavonoids may possess the activity [29].

As far as we know, a lot of sub-groups of flavonoids have been studied for their H1N1 NA-inhibitory activity, including flavones, like apigenin and luteolin; isoflavones, like daidzein and genistein; bioflavonoids, like ginkgetin and hinokiflavone; chalcones; aurones and stilenes, *etc.*, with a range of *IC*_50_ values from 2.2 to 97.1 μM [23,24,25,26]. The relationship between structures and *in vitro* anti-viral activities can be summarized as follows: hydroxyl groups in position 4' and 7, an oxo group in position 4, and a double bond between position 2 and 3 are necessary for good NA inhibition [23,25]. In the present study, flavonoids **1** and **2** showed moderate inhibitory effects against neuraminidase (NA) of influenza virus, with *IC*_50_ values of 29.77 ± 6.12 and 36.91 ± 3.80 μg/mL, respectively. The four flavonoids all significantly inhibited the expression of IL-6 and MCP-1 induced by influenza H1N1 virus at various doses from 3 to 30 μg/mL. This study showed that the skeleton of flavonoids **1**–**4** is favorable to NA inhibition. Among them, the new compound **1**, having an unique E ring between rings B and C, showed the best results both in terms of inhibition of NA and anti-inflammatory effects, which indicated that this extra ring is more favorable for activity than the other functional groups substituted at the same positions.

Thus, two possible pathways might be deduced for their antiviral effects: (1) inhibition of virus release from host cells; (2) reduction of the serious inflammatory cytokine storm induced by the influenza virus infection. Nonetheless, our data did not rule out the possibility that other mechanisms may be involved. Although a complete pharmacokinetic study of flavonoids in *P. clypearia* is needed to further precisely address its bioavailability, the present research partly revealed the mechanisms of *P. clypearia* used as a herbal medicine in the treatment of respiratory tract diseases in China for many years.
